# ‘Well Aware’ Quality Improvement Project: Wellbeing Support Awareness for Psychiatry Resident Doctors at Kent and Medway Mental Health NHS Trust

**DOI:** 10.1192/bjo.2026.11464

**Published:** 2026-06-30

**Authors:** Carolina Pressanto, Suresh Thapaliya, Eric Barratt, Rachel Daly

**Affiliations:** 1 Kent and Medway Mental Health NHS Trust, Maidstone, United Kingdom; 2 Kent and Medway Mental Health NHS Trust, Canterbury, United Kingdom

## Abstract

**Aims:**

This Quality Improvement Project (QIP) aims to evaluate the awareness of wellbeing resources among Core and Higher Psychiatry Resident doctors at Kent and Medway Mental Health NHS Trust. Additionally, it explores barriers to accessing these resources and identifies preferred methods for the delivery of future wellbeing initiatives.

**Methods:**

A collaborative initiative was established between the Medical Education department, the Trust’s Health and Wellbeing team, and resident doctor representatives.

A survey, adapted from a previous QIP, was developed to collect demographic data and training details. It utilized 5-point Likert scales, multiple choice and open-ended questions. The survey was hosted on LimeSurvey and distributed via email to all Core and Higher trainees over a two-month period (October–November 2025).

**Results:**

Twenty-three residents completed the survey. The cohort was predominantly aged 26–35 (87%), female (70%), International Medical Graduates (IMG) (83%), and at Core training level (57%).

Regarding baseline awareness, residents were most familiar with mental wellbeing resources (57%), followed by financial wellbeing and work-life balance (both 35%). Awareness of specific providers included community organizations/charities (43%), local staff wellbeing support (35%), and British Medical Association wellbeing services (35%). While 83% had engaged in a wellbeing conversation with a senior clinician, 91% had not utilized the existing wellbeing resource pack, and 83% had not accessed any formal support services. Participants identified mental wellbeing and work-life balance (both 57%) as the primary areas for future support. Preferred delivery methods included in-person events (78%), email (65%), and induction presentations (43%).

Upon comparing awareness of wellbeing resources, perceived provision of information, and perceived sufficiency of wellbeing offers among groups, we found that Specialty Trainees consistently reported higher mean scores than Core Trainees across all outcome measures; non-IMG doctors reported higher mean scores than IMG doctors across all outcomes; White respondents reported the highest mean scores across all outcomes, while Asian and mixed ethnicity respondents reported lower average awareness and satisfaction.

Qualitative analysis identified “email fatigue”, clinical workload, information access and perceived lack of relevance as significant barriers to engagement.

**Conclusion:**

This project highlights a significant gap between resource availability and trainee utilization. While awareness of mental health support exists, practical barriers such as workload and information overload hinder engagement. The next phase of this QIP will focus on implementing targeted, in-person interventions and streamlining communication to better meet the specific needs of resident doctors.

